# Synthesis, pharmacokinetic studies, and metabolite analysis of meridianin C in rats using a validated ultra high performance liquid chromatography-tandem mass spectrometry method

**DOI:** 10.3389/fphar.2025.1633157

**Published:** 2025-09-09

**Authors:** Liangyu Xu, Zhaogen Wu, Karna Ramachandraiah, Guihun Jiang, Guozhe Zhang

**Affiliations:** ^1^ College Hospital, Jilin Medical University, Jilin, China; ^2^ School of Public Health, Jilin Medical University, Jilin, China; ^3^ Louisiana State University Health Sciences Center, New Orleans, LA, United States; ^4^ Department of Translational Medicine, Jiangsu Medical College, Yancheng, China

**Keywords:** meridianin C, indole alkaloid, synthesis, metabolites, pharmacokinetics, UHPLC-MS/MS

## Abstract

**Introduction:**

Meridianin C (MC) is a marine-derived indole alkaloid that has demonstrated kinase inhibitory and anti-tumor activities. Despite its diverse biological properties, no previous reports have systematically evaluated the in vivo quantitative analysis of MC and its metabolites.

**Methods:**

In this study, MC was synthesized following a previously reported procedure with slight modifications. A sensitive, accurate, and reliable ultra-high performance liquid chromatography-tandem mass spectrometry (UHPLC–MS/MS) method was developed to simultaneously detect MC and its five major metabolites (MC-1-N-O-GluA, MC-1-N-O-SO_3_H, MC-2′-N-O-GluA, MC-2′-N-O-SO_3_H, and MC-O-GluA-didehydration) in rat plasma. Rats received a single oral dose of MC (100 mg/kg), and pharmacokinetic analysis was subsequently performed.

**Results:**

Pharmacokinetic data revealed that MC was rapidly absorbed, with a C_max_ of 44.8 ± 7.0 μmol/L, an AUC_0–48h_ of 232.0 ± 85.9 μmol·h/L, a T_max_ of 0.75 ± 0.27 h, and a t_1/2_ of 17.7 ± 14.1 h. Plasma concentrations of MC were significantly higher than those of its metabolites, suggesting that MC remains the predominant circulating form after oral administration. The identified metabolites mainly resulted from hydroxylation combined with glucuronide conjugation, hydroxylation combined with sulfation, and hydration combined with glucuronide conjugation.

**Discussion:**

These findings demonstrate that the primary metabolic pathway of MC involves hydroxylation (phase I) followed by conjugation (phase II). To our knowledge, this represents the first systematic investigation of the pharmacokinetic characteristics of MC and its metabolites in rats. The study not only advances understanding of MC disposition but also provides a valuable reference for future pharmacokinetic evaluations of other marine-derived indole alkaloids.

## 1 Introduction

In recent years, marine-derived natural products have gained significant attention in pharmaceutical research and development owing to their structural diversity and potent bioactivities (Lu et al., 2025). Marine organisms—including sponges, tunicates, mollusks, marine bacteria, and fungi—have become prolific sources of novel secondary metabolites, many of which possess unique chemical scaffolds and demonstrate promising activities against cancer, infectious, and metabolic diseases. Notably, marine-derived natural products have shown distinct chemical novelty, new mechanisms of action, and enhanced biological activity compared with terrestrial compounds, which contributes to their increasing application in drug discovery and development (Fernández et al., 2024; Singh et al., 2024). Several marine alkaloidal drugs have achieved clinical success worldwide, such as Trabectedin (Yondelis^®^) and Lurbinectedin (Zepzelca^®^), which have been approved for cancer therapy, demonstrating the great translational potential of marine natural products (Molinski et al., 2009; Markham, 2020). Recent advances show that indole-derived marine natural products not only target tumor proliferation but also help overcome multidrug resistance by acting on efflux transporters such as BCRP (Breast Cancer Resistance Protein), providing new therapeutic avenues for resistant malignancies (Kanoujia et al., 2023).

**FIGURE 1:**
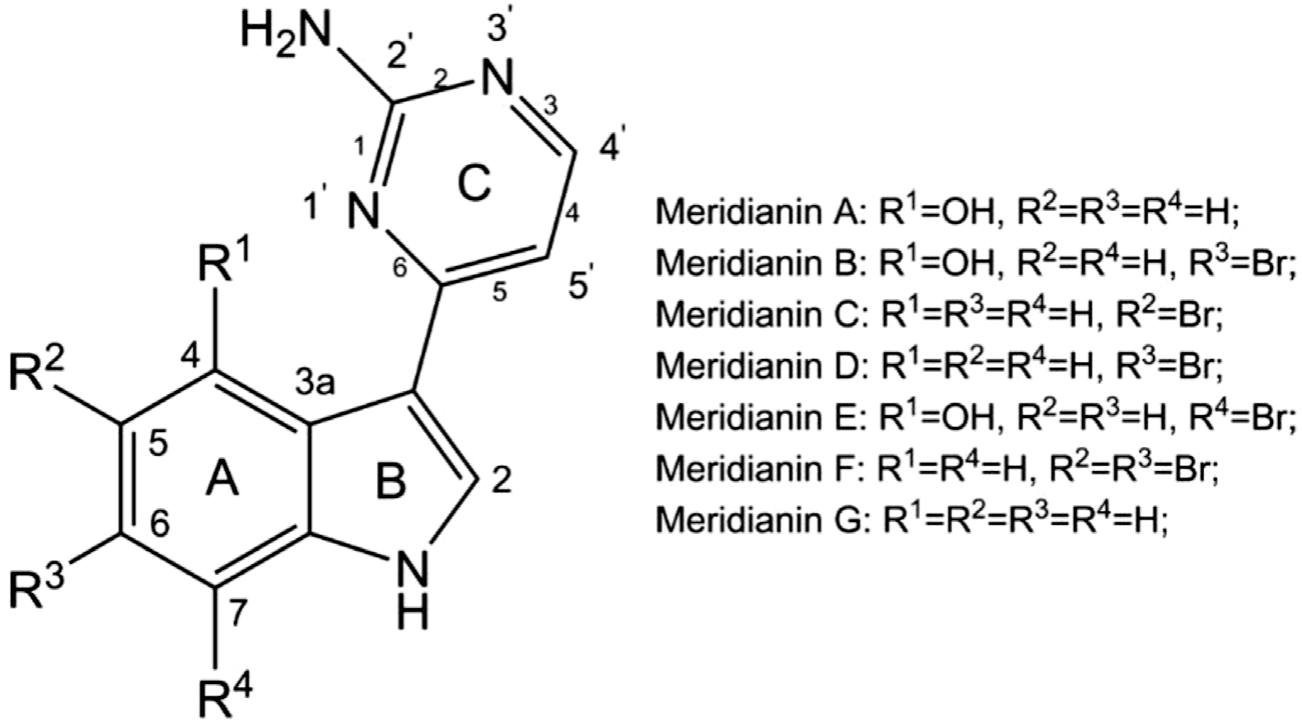
Structure of meridianins **(A–G)**.

Among these marine natural products, indole alkaloids from marine invertebrates, especially ascidians and sponges, have been highlighted as a rich source of lead compounds for drug discovery (Pindur and Lemster, 2001). Meridianins (Figures 1A–G) are a family of brominated indole alkaloids first isolated from the Antarctic tunicate Aplidium meridianum (Gompel et al., 2004; Franco et al., 1998). Particularly, meridianins have received considerable attention due to its potent protein kinase inhibitory activity (More et al., 2014) and a broad spectrum of pharmacological properties, including antitumor (Radwan and El-Sherbiny, 2007), antimalarial (Lebar et al., 2011), anti-Alzheimer’s activity (Llorach-Pares et al., 2017) and antituberculosis effects (Y et al., 2015).

Meridianin C (MC), one of the most prominent members of the meridianin family, is characterized by an indole nucleus substituted at the C-3 position with a 2-aminopyrimidine ring, and a bromine atom at the C-5 position of the indole ring (More et al., 2014). Pharmacologically, MC displays a broad spectrum of bioactivities. It has shown potent inhibition against a range of protein kinases, such as casein kinase 1 (Fernández et al., 2024) and cyclin-dependent kinase 1 (Gompel et al., 2004), implicating its potential as a multi-target kinase inhibitor in cancer therapy and neurodegenerative disease research. In addition, MC has demonstrated strong antiproliferative effects against human tongue cancer cell lines (Park et al., 2018), as well as improved glucose uptake via GSK-3β inhibition (Han et al., 2021), supporting their therapeutic prospects not only in oncology but also in metabolic diseases such as diabetes.

Given these remarkable activities, MC has frequently served as a lead compound for the design of novel small molecules (More et al., 2014; Han et al., 2021), similar to other natural anticancer agents such as ferulic acid, isothiocyanates and apigenin (Liu et al., 2025; Guo et al., 2025; Liang et al., 2023). Numerous synthetic MC derivatives have demonstrated improved anti-proliferative and kinase inhibitory properties compared to the parent compound (More et al., 2014; Han et al., 2021; Cho et al., 2020). In addition, Han et al. reported MC analogues with superior glucose uptake activity to MC through GSK-3β inhibition in hepatic cells (Han et al., 2021), while Park et al. demonstrated anti-adipogenic effects of MC derivatives by downregulating key transcription factors and adipokines (Park et al., 2014).

Despite these advances, most research has focused on the synthesis, structural modification, and *in vitro* biological evaluation of MC and its analogues. However, their pharmacokinetic profiles, metabolic fate *in vivo* and druggability remain inadequately explored. As pharmacokinetic studies are essential for understanding the absorption, distribution, metabolism, and excretion (ADME) properties of drug candidates (Moda et al., 2008), the lack of comprehensive *in vivo* evaluation greatly limits the translational potential of MC.

**FIGURE 2:**
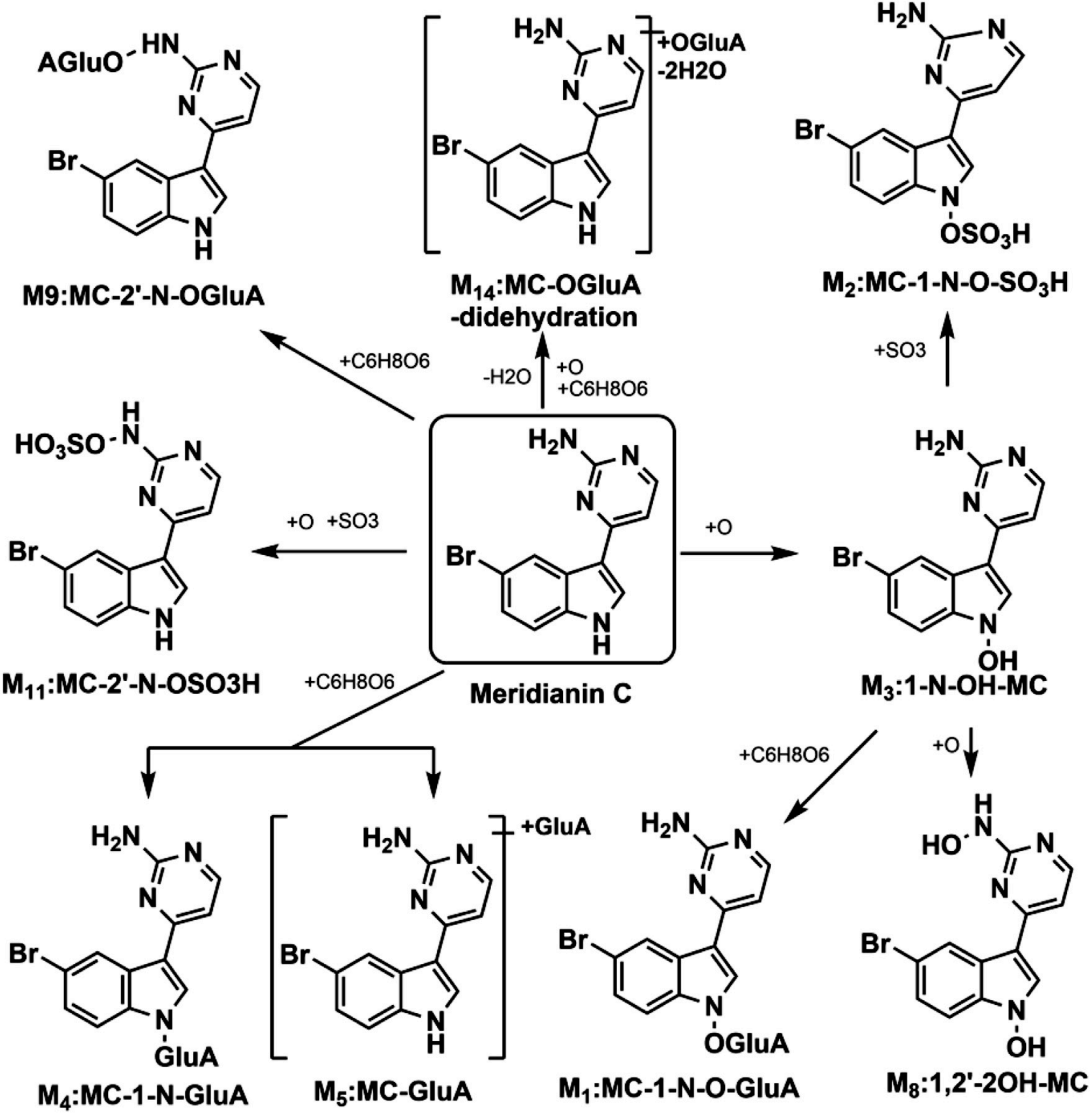
Proposed metabolic routes of MC in rat plasma (GluA indicates glucuronidation).

Previously, our group characterized nine metabolites of MC in rat plasma using UHPLC/Q-TOF MS (Table 1), which enabled semi-quantitative analysis based on relative signal intensities (Zhang et al., 2021). The proposed metabolic pathways of MC, as illustrated in Figure 2, were established in our previous research. While this approach is valuable for identifying metabolites and elucidating metabolic pathways, it does not provide accurate or validated quantification of MC and its metabolites due to limitations in sensitivity, specificity, and reproducibility. In contrast, this study is the first to develop and validate a UHPLC-MS/MS method for the absolute quantification of MC and its five major metabolites in rat plasma, enabling comprehensive and reliable pharmacokinetic analysis *in vivo*. The UHPLC-MS/MS technique offers significant advantages, including superior sensitivity, specificity, and reproducibility, making it ideal for precise quantification in complex biological matrices (Yang et al., 2022; Hao et al., 2026; Cui et al., 2022). Additionally, MC was synthesized using a modified protocol (Huggins et al., 2018) to support this investigation. Overall, this advancement provides critical data to support the preclinical evaluation and drug development of MC.

**TABLE 1:** Qualitative analysis of MC and its metabolites in rat plasma by UHPLC/Q-TOF MS.

RT (min)	NO.^a^	Calculated Mass^b^	Observed mass (Da) Fragment ions	Error (mDa)	Type of metabolite	Formula
2.49	**M1**	481.0359	481.0365/483.03,305.00/307.00,226.09,185.07	−0.6	Hydroxylation + glucuronide conjugation	C_18_H_17_BrN_4_O_7_
2.97	**M2**	384.9606	384.9613/386.96,305.00/307.00,226.09,209.06,197.08,185.07	−0.7	Hydroxylation + sulfation	C_12_H_9_BrN_4_O_4_S
3.24	M3	305.0038	305.0118/307.01,289.01,226.09,185.07	−8.0	Hydroxylation	C_12_H_9_BrN_4_O
3.33	M4	465.0410	465.0421/467.0406	−1.1	Glucuronide conjugation	C_18_H_17_BrN_4_O_6_
3.38	M5	465.0410	465.0416,305.00/307.00,226.08,210.09,185.07,169.08	−0.6	Glucuronide conjugation	C_18_H_17_BrN_4_O_6_
3.78	M8	320.9987	320.9985/322.9968	0.2	2 × Hydroxylation	C_12_H_9_BrN_4_O_2_
3.85	**M9**	481.0359	481.0359/483.03,305.00/307.00,226.09,209.06,198.09,143.06	0.0	Hydroxylation + glucuronide conjugation	C_18_H_17_BrN_4_O_7_
4.22	**M10**	289.0089	289.0089/291.01,209.08,169.08,140.05	0.0	Parent drug	C_12_H_9_BrN_4_
4.37	**M11**	384.9606	384.9608/386.96,305.00/307.00,226.09,209.06,198.09,185.07 143.06	−0.2	Hydroxylation + sulfation	C_12_H_9_BrN_4_O_4_S
6.06	**M14**	445.0148	443.0180/445.0161,288.00/290.00,246.99/248.99,208.07,168.07	−1.3	Hydration + glucuronide conjugation +2 × Alcohols dehydration	C_18_H_13_BrN_4_O_5_

^a^ Metabolites in bold font were quantified.

^b^ Only one molecular ion of the isotopes was shown.

^*^ Bold values indicate the prototype compound (M10) and its five metabolites in the pharmacokinetic study.

## 2 Materials and methods

### 2.1 Chemicals

Puerarin (purity >98%) as internal standard (IS) was purchased from Chengdu Herbprify CO. LTD. (Beijing, China). HPLC-grade acetonitrile and formic acid were bought from Thermo Fisher Scientific (Boston, MA). Ultrapure water was obtained by a Milli-Q water purification system (Millipore, France). All other chemicals were of analytical grade.

### 2.2 Synthesis of MC

**FIGURE 3:**
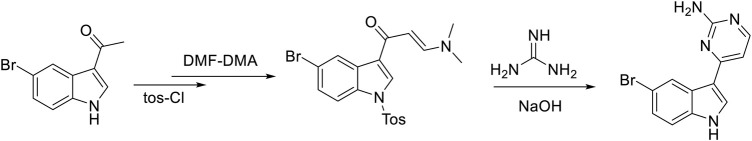
Synthesis of MC.

As depicted in Figure 3, MC was synthesized in four steps starting from 5-bromoacetyl indole, and the nitrogen of raw material was then protected using p-toluenesulfonyl chloride (TsCl), triethylamine, and 4-dimethylaminopyrimidine (DMAP) in a solvent (DCM). The enaminone was then obtained by reacting protected in-dole derivative with DMF/dimethylformamide-diemthylacetal (DMF-DMA) at reflux for 5 h. The reaction of cyclization and deprotection of the enaminone derivative were completed by utilizing potassium carbonate and guanidine hydrochloride to finally synthesize MC (Huggins et al., 2018).

### 2.3 Experimental animals

Sprague Dawley rats (male, 220–250 g) were purchased from the SPF Biotechnology Co., Ltd. (Beijing, China). Animals were acclimated under controlled conditions (temperature 22 °C ± 2 °C, humidity 50%–60%, 12 h light cycle) with unlimited fodder and water access for 7 days. The animal experiments were approved by the Animal Experiment Ethics Review Committee of Jiangsu Vocational College of Medicine, Yancheng, China (LLSQ-2021-031106).

### 2.4 Drug administration and sampling

After fasting overnight, six rats were orally administered 100 mg/kg MC suspended in 0.5% carboxymethyl cellulose sodium solution (10.0 mg/mL). Blood samples (about 300 μL) were collected into heparinized polythene tubes from the suborbital vein at 0, 0.25, 0.5, 1, 2, 3, 4, 8, 12, 24, 36 and 48 h after administration. All samples were immediately centrifuged at 6,125 *g* for 5 min at 4 °C to acquire plasma. The plasma samples were preserved at − 80 °C until further analysis.

### 2.5 Preparation of calibrations samples and quality control samples

MC, accurately weighted, was firstly dissolved in methanol to acquire the standard stock solutions (250 μg/mL). A set of standard working solutions (25,000.0 ng/mL, 12,500.0 ng/mL, 2,500.0 ng/mL, 1,250.0 ng/mL, 625.0 ng/mL, 250.0 ng/mL, 125.0 ng/mL, 31.25 ng/mL) were obtained by diluting the stock solutions with methanol. An aliquot of 20 µL of these standard working solutions was then spiked into 1.5 mL polythene tubes and the solvent of methanol was evaporated at room temperature. Afterwards, the calibration standards (5,000.0 ng/mL, 2,500.0 ng/mL, 500.0 ng/mL, 250.0 ng/mL, 125.0 ng/mL, 50.0 ng/mL, 25.0 ng/mL, 6.25 ng/mL) were obtained by adding blank plasma (100 µL) into the tubes and fully mixing. The preparation method of quality control samples (QCs, 25.0 ng/mL, 1,250.0 ng/mL, 4,000.0 ng/mL) was identical with that of the calibration standards. All solutions were stored at 4 °C until further use.

### 2.6 Samples processing

For the measurement of the concentrations of MC and its metabolites, three times volume of methanol, containing 20 µL IS (puerarin, 2,160.0 ng/mL), were added to 100 µL plasma samples to precipitate protein. After vortexing for 2 min, the mixtures were centrifuged at 6,125 *g* for 10 min at 4 °C. The obtained supernatants were transferred to fresh tubes and allowed to be dried at room temperature by a nitrogen blowing instrument. Then, the remaining residues were re-dissolved with 100 µL methanol–water (80:20, v/v). Following centrifugation at 16,173 *g* for 10 min, the supernatants were transferred and used for UHPLC/Q-TOF MS assessment. The injection volume of each sample was 1 μL.

### 2.7 UHPLC and MS conditions

The UHPLC-MS system comprised of an Acquity UPLC system (Waters, Milford, United States) and Waters TQD tandem mass spectrometer equipped with an electrospray ionization (ESI) source. A Waters ACQUITY BEH C18 column (100 × 2.1 mm, 1.7 μm, Waters, Milford, United States) was used for the chromatography separation. The column temperature was set to 40 °C. The mobile phase was composed of 0.1% formic acid in water (A) and 0.1% formic acid in acetonitrile (B) with a fixed flow rate at 0.4 mL/min. A gradient elution program was used as follows: 0–1.5 min, 5%–20% B; 1.5–5 min, 20%–30% B; 5–7 min, 30%–95% B; 7–9 min, 95% B; 9–11 min, 95% B.

**FIGURE 4:**
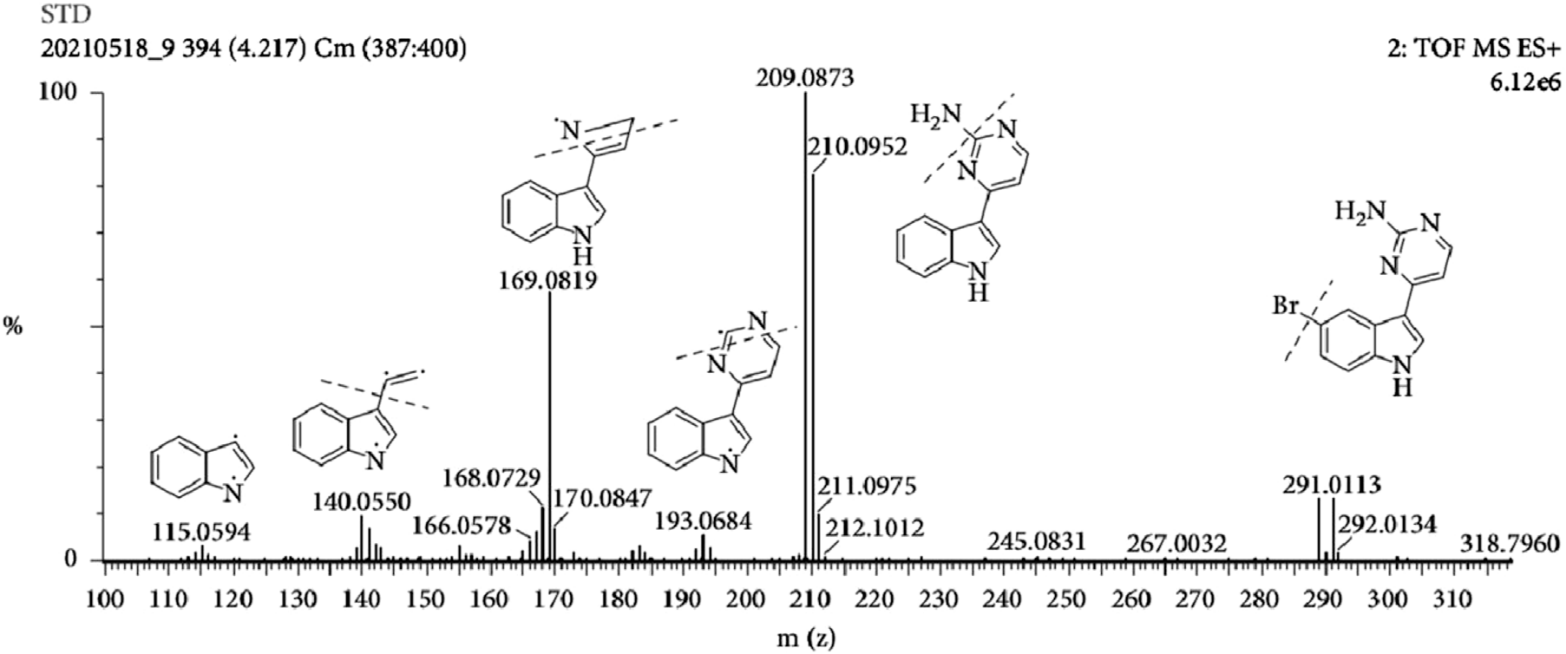
The mass spectrum of MC and the chemical structures of its MS/MS fragments.

**FIGURE 5:**
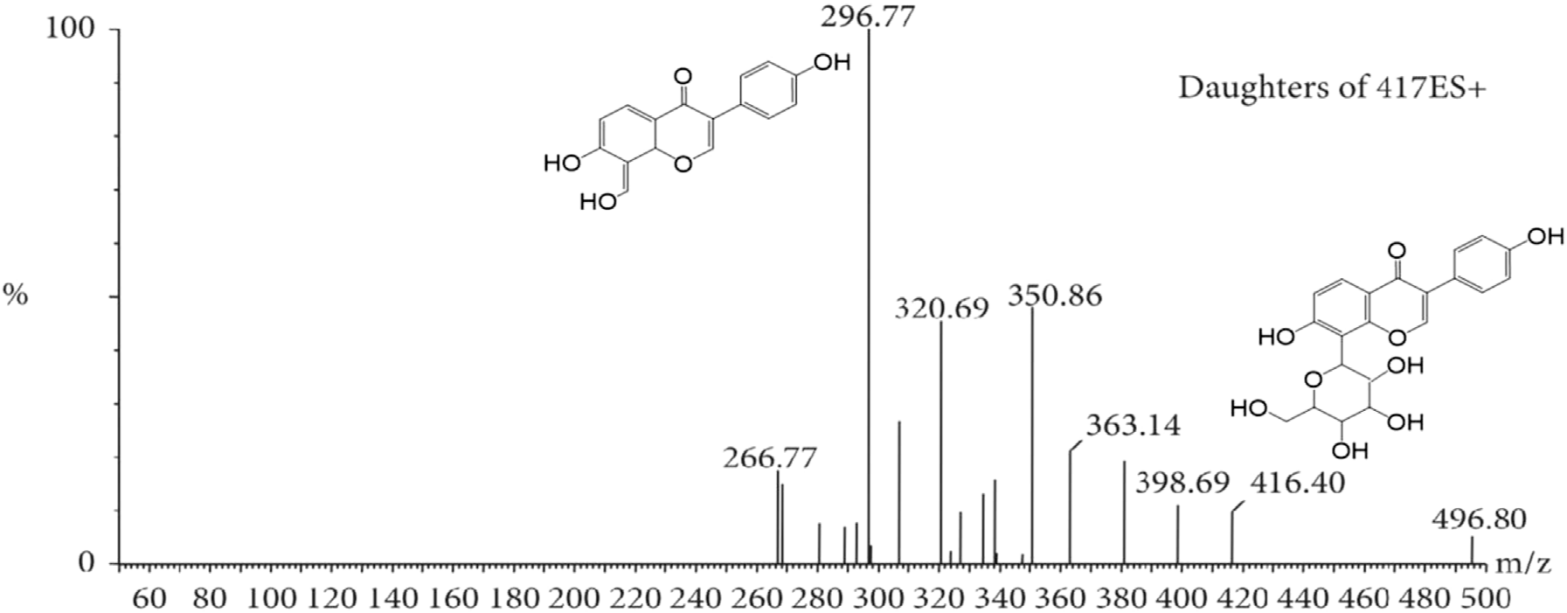
The mass spectrum of puerarin and the chemical structures of its MS/MS fragments.

The MS tune parameters were set as mentioned below. The desolvation of the source temperatures was set at 500 °C. The flow rate of nebulization gas was 650 L/h. The capillary voltage was set at 2.8 kV in the positive ionization mode. The cone voltages were maintained at 35 V for all the tested ingredients. All data were obtained via the multiple reaction monitoring (MRM) ion mode. The range of m/z for the data collection was set at 100–1,500 Da. Transitions, cone voltage and a collision energy of MC and its metabolites were optimized (Table 2). The mass spectrum of MC and the chemical structures of its MS/MS fragments is shown in Figure 4, and The mass spectrum of puerarin and the chemical structures of its MS/MS fragments is shown in Figure 5. MassLynx™ software version 4.1 was used for data acquisition and TargetLynx™ (Waters, Milford, MA, United States) was applied to quantitation.

**TABLE 2:** Transitions, cone voltage and a collision energy of compounds in MRM mode.

Compounds	Transitions	Collision energy (V)	Cone voltage (V)
M1, M9	483.0→305.0	42	35
M2, M11	385.0→226.1	42	35
MC	289.0→209.1	45	35
M14	443.0→288.0	45	35
PUR (IS)	417.1→297.0	30	35

### 2.8 Method validation

All method validation procedures were performed strictly in accordance with the US FDA Guidance for Industry on Bioanalytical Method Validation (FDA, 2018). The assessment of selectivity was performed via comparing chromatograms among blank rat plasma (six different batches) and rat plasma spiked with MC and IS. The evaluation of linearity was carried out using weighted (1/x^2^) least-squares analysis of two sets of calibration standards. Three different concentrations of Quality Check: QCs (n = 6) standards were used and detected on three different days to evaluate the intra-day and inter-day precision and the accuracy. Similarly, matrix effect and extraction recovery were assessed using three different concentrations of QCs (n = 6). The matrix effect was evaluated via calculating the peak area ratio of MC in post-spiked QCs and the solvent-substituted samples with same concentration. The extraction recovery was assessed via calculating the peak area ratio of MC in pre-spiked and post-spiked QCs. The stability of MC was examined using three concentrations of QCs (n = 3) under three different storage conditions, including freeze-thaw stability (three cycles from −20 °C to ambient temperature), short-term stability (storage at 20 °C–25 °C for 24 h), and long-term stability (storage at −20 °C for 30 days).

### 2.9 Pharmacokinetics study

The concentrations of MC in the collected plasma samples were quantified by the established calibration curve based on peak areas of corresponding components. Owing to the lack of corresponding standards, calibration curves for the metabolites were not established. It has been reported that the concentrations of the metabolites could be quantified according to calibration curve of parent drug. This is possible due to the structural similarity of their parent nucleus (Zhang et al., 2015). Therefore, in this study, the concentrations of the metabolites were calculated via the calibration curve of MC. The main pharmacokinetic parameters of MC and its metabolites were calculated via a non-compartmental model with the pharmacokinetic software, DAS (ver. 2.0, Chinese Pharmacology Association, Shanghai, China).

### 2.10 Data analysis

Pharmacokinetic parameters were evaluated by DAS software (version 2.0, Chinese Pharmacological Society, Shanghai, China), including the area under the plasma concentration-time curve (AUC_0–t_), time to reach the maximum plasma concentration (T_max_), elimination half-time (t_1/2_), the maximum plasma concentration (C_max_). Data were expressed in mean and standard deviation (SD) of each group. A paired t-test was used to analyze the significance of the differences between the two groups. p-value was considered statistically significant if p < 0.05 (SPSS 18.0 software, SPSS Inc., Chicago, IL, United States).

## 3 Results

### 3.1 Synthesis of MC

Synthesis of MC was performed as described by Huggins et al. (2018) with slight modifications. MC was synthesized in four steps as depicted in Figure 3. The reactions were monitored by TLC. The synthesis of MC was achieved with an overall yield of 32%. The structure of MC was confirmed by both 1H NMR spectrometry (Supplementary Figure S1) and MS techniques (Supplementary Figure S2).

### 3.2 Method validation

#### 3.2.1 Selectivity

**FIGURE 6:**
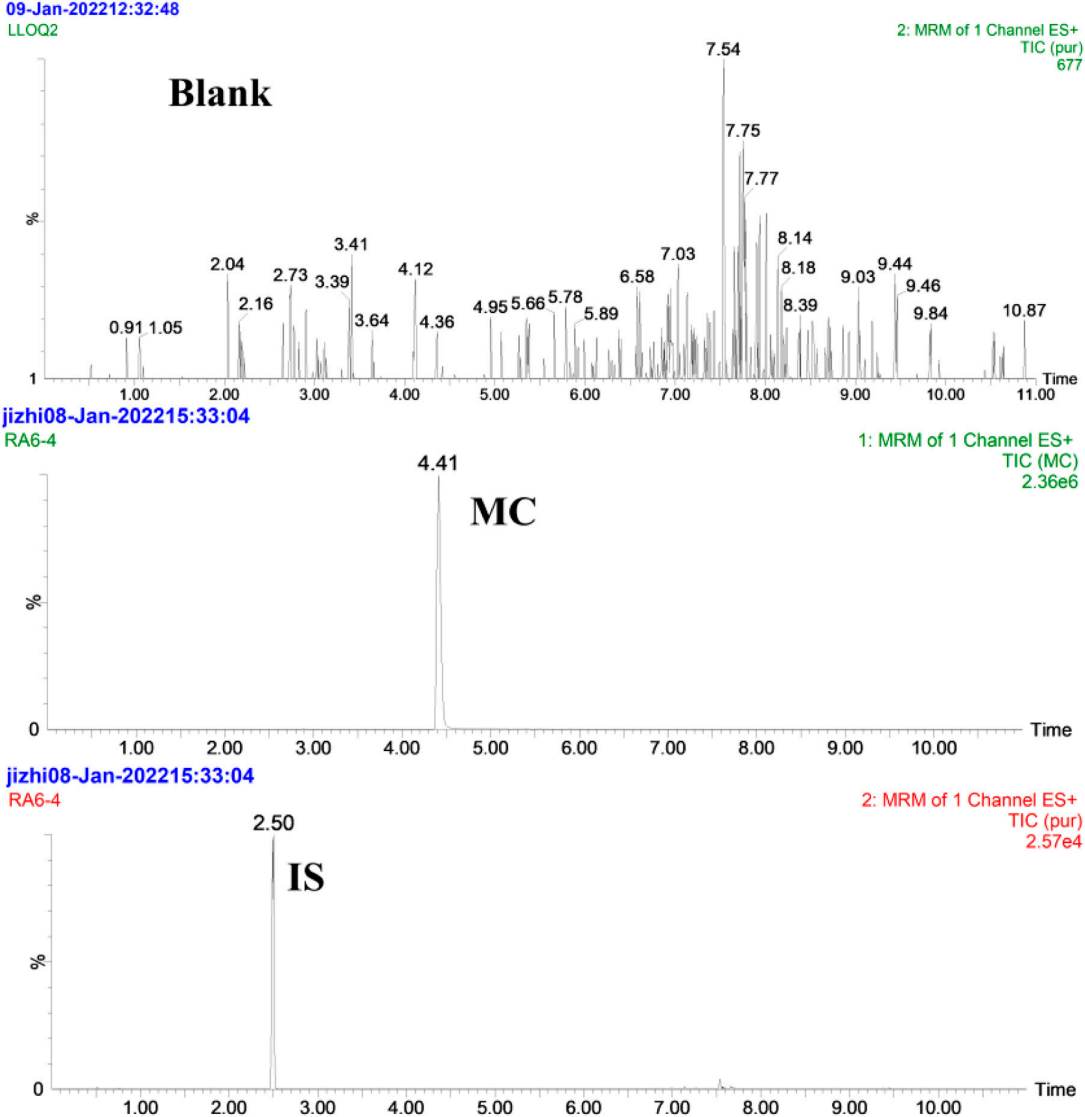
MRM chromatograms of MC and IS in rat plasma; Blank indicates the control blank plasma; MC and IS indicate blank plasma spiked with MC and IS.

**FIGURE 7:**
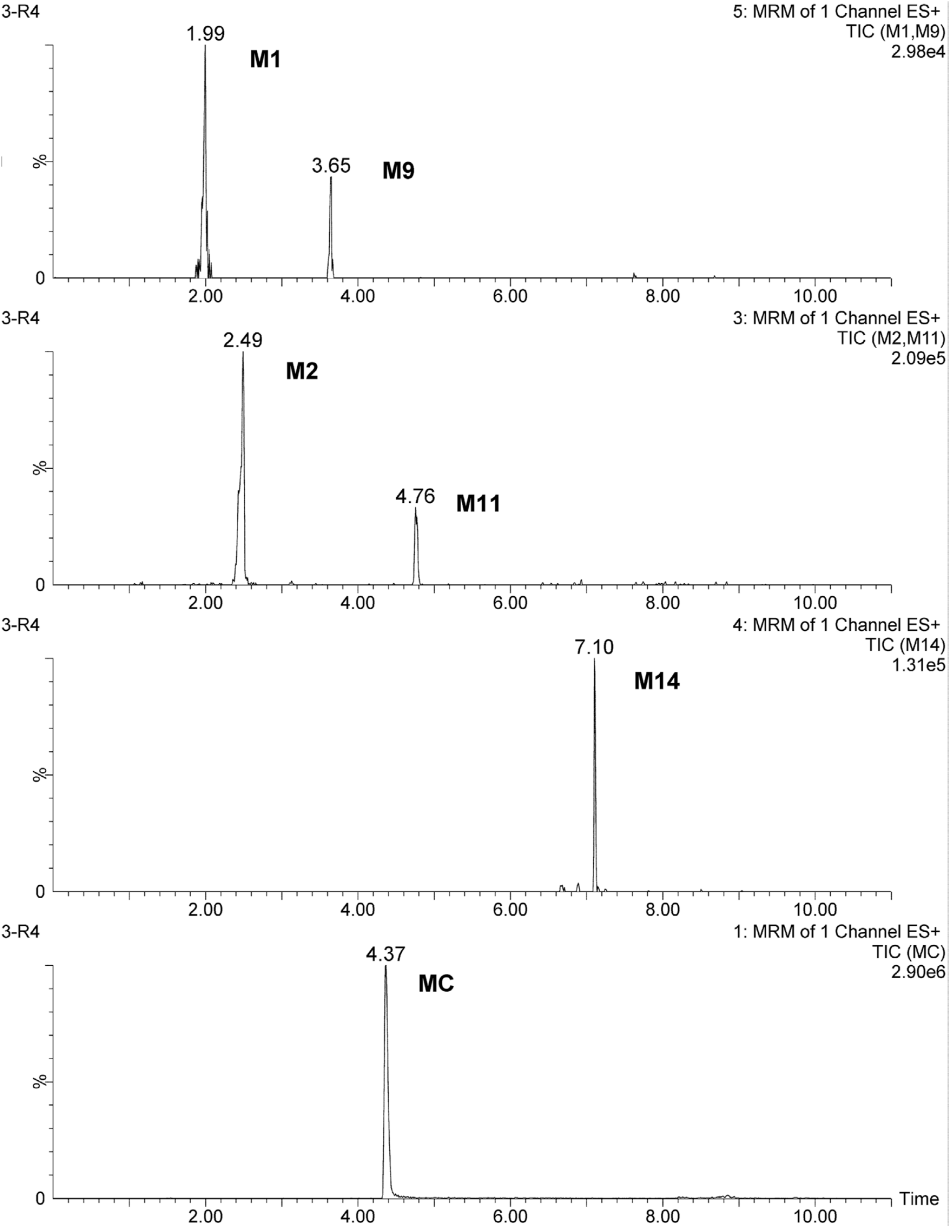
MRM chromatograms of the quantified metabolites in rats at 1 h after oral administration at a dose of 100 mg/kg.

The selectivity of the method was demonstrated by analyzing six different batches of blank rat plasma and rat plasma spiked with MC and Internal Standard (Puerarin). The Multiple reaction monitoring (MRM) chromatograms of blank (control), MC and IS are displayed in Figure 6 while MRM of MC and quantified metabolites are shown in Figure 7. The results showed that there was no obvious endogenous interference in the detection of the analytes and the IS, demonstrating the selectivity of the method.

#### 3.2.2 Linearity and sensitivity

To evaluate the linearity, calibration curve for MC was established by analyzing the spiked calibration samples with the concentrations ranging from 6.25 to 5,000 ng/mL. The obtained equation of the calibration curve was y = 0.0374x + 80.251 (*r* ^2^ = 0.9958), where y represents the peak area ratio of MC/IS and x represents the concentration of MC in rat plasma. The correlation of determination (*r* ^2^) surpassed 0.99, indicating a good linearity of the calibration curve in the measured concentration range. The lower limit of quantifications (LLOQ) of MC in rat plasma was detected to be 6.25 ng/mL. This indicated the higher sensitivity of the system.

#### 3.2.3 Accuracy and precision

To evaluate the precision and accuracy, six replicates of QC samples were detected at three concentrations on three successive days. As shown in Table 3, the range of intraday and inter-day precision values for MC were 7.4%–12.5% and 10.9%–14.3%, respectively. These values were within the limits (<15%). Furthermore, the accuracy values were found to be between −10.5% and 3.2%, which met the criteria of no more than ±15%. The results indicated the optimal reproducibility of the method.

**TABLE 3:** Precision and accuracy of MC in rat plasma (n = 6).

Marker compounds	Concentration (ng/mL)		RSD (%)		RE (%)
	Added	Found	Intra-day	Inter-day	
MC	25.0	25.8	9.8	10.9	3.2
	1,250.0	1,137.5	7.4	11.6	−9.0
	4,000.0	3,580.0	12.5	14.3	−10.5

#### 3.2.4 Recovery and matrix effect

The pre-spiked QCs were obtained by the utilization of methanol, which caused the precipitation of proteins in plasma after the addition of MC and IS. On the other hand, for post-spiked QCs, methanol (protein precipitation) was added into plasma before the addition of MC and IS. The extraction recovery was described as the peak area ratio of MC in pre-spiked and post-spiked QCs. The recovery of MC ranged from 95.9% to 106.1% (Table 4), which complied with the criteria of Chinese Pharmacopoeia. The matrix effect was assessed by calculating the peak area ratio of MC in post-spiked QCs and the sol-vent-substituted samples. Nonetheless, the matrix effect of MC ranged from 94.0% to 108.9% (Table 4), which was within the limits of acceptability.

**TABLE 4:** Recovery and matrix effects of MC and IS in rat plasma (n = 6).

Marker compounds	Concentration (ng/mL)	Recovery (%)	RSD (%)	Matrix effect (%)	RSD (%)
MC	25.0	102.3	2.2	94.0	7.0
	1,250.0	95.9	2.8	108.9	1.9
	4,000.0	106.1	4.6	108.2	2.9
IS	432.0	98.1	6.8	91.8	7.2

#### 3.2.5 Stability

The stability was estimated by analyzing QCs (25.0, 1,250.0, and 4,000.0 ng/mL) after storing in three different conditions: three freeze-thaw cycles (−20 °C to ambient temperature), 20 °C–25 for 24 h, and - 20 °C for 30 days. As presented in Table 5, the deviations in the stabilities of MC ranged from - 8.5% to 13.5% under the three different storage conditions. The results revealed that MC was stable in all the three conditions.

**TABLE 5:** Stability of MC in rat plasma (n = 3).

Marker compounds	Concentration (ng/mL)	Stability (re, %)		
		Freeze-thaw (3 cycles)	20 °C–25 °C/24 h	−20 °C/30 days
MC	25.0	13.5	12.5	10.6
	1,250.0	12.6	7.9	5.2
	4,000.0	9.3	−6.8	−8.5

#### 3.2.6 Pharmacokinetic application

**FIGURE 8:**
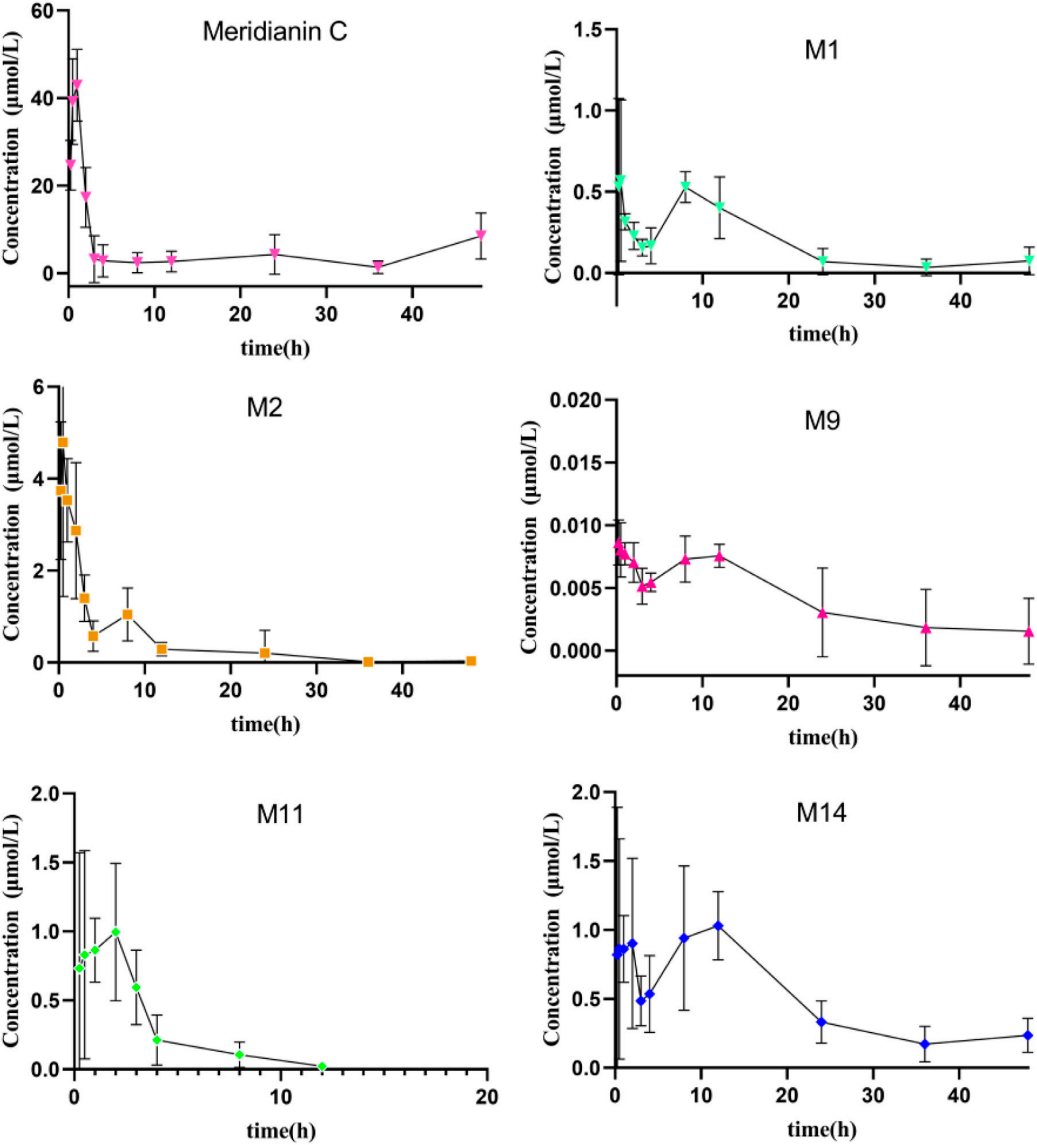
Mean plasma concentration-time curves of MC and its five metabolites in rat plasma after intragastric administration of MC at a single dose of 100 mg/kg (n = 6).

The established UHPLC-MS/MS approach was successfully employed to evaluate the pharmacokinetics of MC and its metabolites (M1, M2, M9, M11 and M14) after a single (dose) oral administration of MC (100 mg/kg) to rats. The mean plasma concentration-time curves of the MC and its metabolites are presented in Figure 8. The main pharmacokinetic parameters estimated via non-compartmental analysis are compiled in Table 6. The C_max_ for MC was 44.8 μmol/L, whereas it’s AUC_0–48h_, T_max_, and half-life (t_1/2_) were found to be 232.0 μmol h/L, 0.75 h, and 17.7 h, respectively. On the other side, the C_max_ for M1, M2, M9, M11 and M14 was 0.92, 6.4, 0.01, 1.6 and 1.9 μmol/L and their T_max_ occurred at 2.5, 0.50, 2.4, 0.88 and 3.9 h, respectively. Additionally, the AUC_0–48h_ of these five metabolites (M1, M2, M9, M11, M14) was estimated to be 27.7, 11.1, 1.3 × 10^3^, 58.7 and 9.9-fold less than that of MC, respectively. However, the system failed to quantify the concentration of M3, M4, M5, and M8 in plasma samples because their contents were lower than the limit of quantification (LOQ).

**TABLE 6:** Pharmacokinetic parameters of MC and its main metabolites in rat plasma after intragastric administration of 100 mg/kg MC (n = 6).

Parameters	Units	MC	M1	M2	M9	M11	M14
AUC_0–48h_	μmol·h/L	232.0 ± 85.9	8.4 ± 2.9	20.9 ± 8.5	0.18 ± 0.12	4.0 ± 1.1	23.4 ± 2.5
*C*_max_	μmol/L	44.8 ± 7.0	0.92 ± 0.47	6.4 ± 2.0	0.010 ± 0.001	1.6 ± 0.6	1.9 ± 0.6
*T*_max_	h	0.75 ± 0.27	2.5 ± 4.7	0.50 ± 0.27	2.4 ± 4.7	0.88 ± 0.63	3.9 ± 5.0
t_1/2_	h	17.7 ± 14.1	6.4 ± 4.3	4.4 ± 2.7	13.5 ± 6.0	3.4 ± 2.2	13.5 ± 7.2

## 4 Discussion

MC, an indole alkaloid derived from marine organisms, is known to possess a variety of pharmacological activities and is often used as a core scaffold for the synthesis of active substances (More et al., 2014; Han et al., 2021). Several studies have proposed MC as a promising lead compound for drug development, but most previous reports have focused on synthesis, structural modification, and *in vitro* biological evaluation (Han et al., 2021). To date, there have been no studies providing validated quantitative data on the pharmacokinetics of MC and its metabolites *in vivo*. Previous investigations—including our own UHPLC/Q-TOF MS metabolite profiling—were limited to semi-quantitative analyses, lacking the sensitivity, specificity, and reproducibility required for robust pharmacokinetic evaluation (Zhang et al., 2021). In contrast, the present study is the first to employ a validated UHPLC-MS/MS method for the simultaneous absolute quantification of MC and its major metabolites, enabling a more comprehensive understanding of their pharmacokinetic profiles *in vivo*.

In our previous study, nine metabolites of MC were detected in rat plasma after oral administration (Zhang et al., 2021). To further investigate their pharmacokinetics profile, we determined the concentrations of MC and its five major metabolites in rat plasma according to the established UHPLC-MS/MS approach (Figure 8). The pharmacokinetic evaluation per-formed by non-compartmental analysis is shown in Table 6. Pharmacokinetic evaluation showed rapid absorption of MC, as indicated by the high plasma concentrations of MC and its metabolites at 0.75 h post-dosing. This is consistent with findings by Kushida et al. (2021), who reported that indole alkaloids in Uncaria Hook (e.g., geissoschizine methyl ether) are rapidly absorbed into the bloodstream after oral administration, with a T_max_ of 0.42–0.67 h. The C_max_ and AUC_0–48h_ of MC were significantly higher than those of its metabolites, indicating it remains the predominant plasma compound post-oral administration—suggesting favorable bioavailability. This contrasts with Hase et al.’s findings on vincamine (a structurally related indole alkaloid): they noted vincamine has low bioavailability, further affected by formulation and other components (Hasa et al., 2013). In our study, MC maintains high plasma levels in its original form with high Cmax and AUC_0–48h_, pointing to better bioavailability than vincamine and potentially other related indole alkaloids. Additionally, the observed multiple peaks in the plasma concentration–time profile and the long half-life (17.7 ± 14.1 h) imply possible enterohepatic recirculation, a phenomenon also documented for other indole alkaloids such as 10-methoxycamptothecin (Shao et al., 2017). Enterohepatic recirculation has been shown to enhance drug exposure and prolong the pharmacological effects of various natural products (Gao et al., 2014).

It was also observed that M2 (MC-1-N-O-SO_3_H) and M14 (MC-O-GluA-didehydration) were the two metabolites with relatively high AUC_0-48h_ values. This showed that hydroxylation + sulfation and hydroxylation + glucuronide conjugation were the major metabolic pathways (phase II metabolism) of MC, which is consistent with the findings of our previous study (Zhang et al., 2021). This phase II metabolism pattern aligns with previous reports on the metabolism of other natural products, where conjugation typically increases polarity and reduces toxicity (Lentini et al., 2020). Importantly, some glucuronidated or sulfated metabolites may retain or even surpass the biological activities of the parent compound, as reported for morphine-6-glucuronide and berberrubine-9-O-β-D-glucuronide (Klimas and Mikus, 2014; Yang et al., 2017). Given the broad bioactivity spectrum of MC, future studies should focus on evaluating the pharmacological potential of its glucuronidated metabolites, which may provide improved efficacy and safety.

## 5 Conclusion

In summary, this study is the first to quantitatively analyze Meridianin C (MC) and its major metabolites *in vivo*, providing validated pharmacokinetic data. A sensitive and validated UHPLC-MS/MS method was developed for the simultaneous quantification of MC and its five major metabolites in rat plasma, and this rapid method can be readily extended to other halogenated indole alkaloids. Pharmacokinetic analysis demonstrated that MC had higher systemic exposure (AUC_0_–_48_h) than its metabolites. Notably, metabolites M2 and M14, which exhibited relatively high AUC_0_–_48_h values, revealed that hydroxylation combined with sulfation and hydroxylation combined with glucuronidation are the primary phase II metabolic pathways. These findings highlight the significance of phase II metabolites in drug development and provide valuable data for the preclinical development of MC-based drug candidates. Future studies should investigate the *in vivo* pharmacological activity of these metabolites and further characterize the pharmacokinetics and tissue distribution of MC to fully elucidate its druggability and therapeutic potential.

## Data Availability

The original contributions presented in the study are included in the article/Supplementary Material, further inquiries can be directed to the corresponding authors.
