# Supplementary material for: Synthesis, pharmacokinetic studies, and metabolite analysis of meridianin C in rats using a validated ultra high performance liquid chromatography-tandem mass spectrometry method
Source: Front Pharmacol. 2025 Sep 9;16:1633157. doi: 10.3389/fphar.2025.1633157 (PMC12455078; doi:10.3389/fphar.2025.1633157)
Supplement: Supplementary file 1 [file DataSheet1.docx]

Supplementary Material

# Supplementary Figures


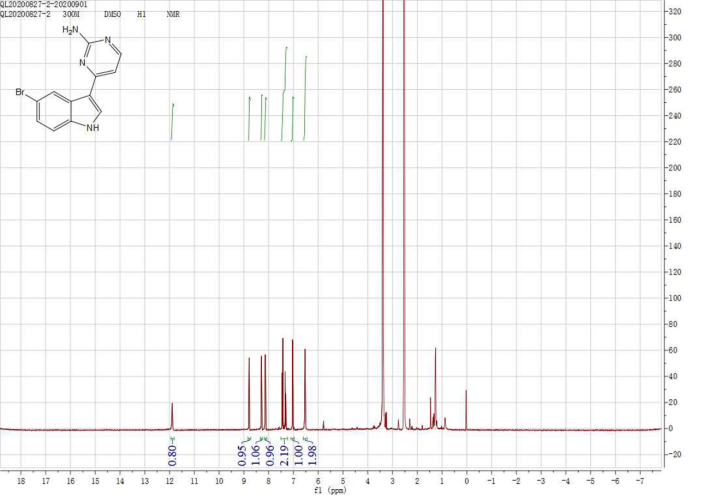


**Supplementary Figure 1.** 1H-NMR spectra of MC.


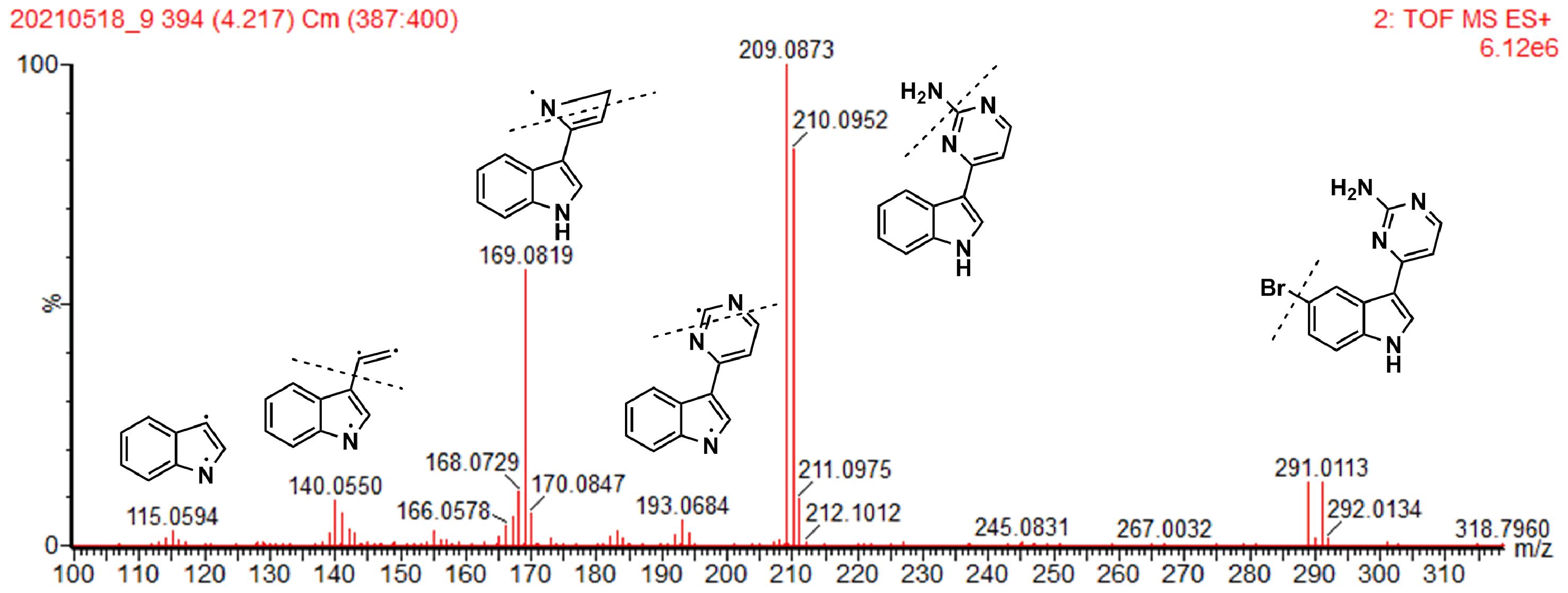


**Supplementary Figure 2.** The mass spectrum of MC.
